# Nucleoproteid in Urine

**Published:** 1907-08-17

**Authors:** 


### Clinical Points.

### NUCLEOPROTEID IN URINE.

When, after boiling the upper part of a specimen of urine in a test tube, acetic acid is added to clear up any phosphates that may have come down, a faint residual cloud is sometimes seen. Such a cloudiness may be due to albumen, but it is most important to remember that it may also be due to nucleoproteid. If this be so, the same specimen of urine will give no white ring with nitric acid. If the medical examiner reports to an insurance company that there is slight albuminuria, the risk upon a proposer's life will very likely be declined; whereas if the report is that the urine is free from albumen, it would probably be accepted at tabular rates. Upon so small a matter, therefore, as the interpretation of a faint cloud given by the boiling test very serious issues may depend.

The reason why nucleoproteid causes this difficulty in testing for albuminuria is that it is precipitated by acetic acid. It is not precipitated by nitric acid; indeed, a cloud due to nucleoproteid is re-dissolved by a drop of nitric acid, which is a point made use of in distinguishing albumen from nucleoproteid.

The Significance of Nucleoproteid in Urine.

When urines are sent to clinical laboratories for general examination the tests applied are so carefully performed that small things are reported about the specimen, the clinical significance of which is often difficult to interpret. It again and again happens, for example, that a report comes back thus:

*Albumen.*—No albumen is present in this specimen, but there is a very distinct trace of nucleoproteid.

The questions at once arise:—What is the importance of the presence of nucleoproteid in this urine? Is it a dangerous condition? Does it mean anything worth troubling about? These questions are constantly being asked. The answers are as follows:—

Nucleoproteid, when carefully tested for, is present in a very large number of normal urines. Many, of course, contain no nucleoproteid at all; but in more urines than not a distinct trace can be found by the acetic acid test. The report might well read: "A *normal* trace of nucleoproteid is present." The significance as regards the patient is nil; the chief importance of the fact lies in the difficulty there may be in distinguishing the nucleoproteid from albumen.

On the other hand, a urine should never contain more than a trace of nucleoproteid; if the report is to the effect that "this specimen contains excess of nucleoproteid," there is always some other abnormality as well. The nucleoproteid in itself is not a sign of danger, nor does it, like albumen, suggest Bright's disease as possible or likely; but when excess of it is present there is usually something irritating the pelves of the kidney, the ureters, bladder, or urethra. Microscopical examination of the centrifugalised deposit from the urine will nearly always detect the nature of this irritation, and if there is one thing more than another that the presence of excess of nucleoproteid in a urine indicates it is the necessity for a microscopical examination of the deposit. Unsuspected pyuria, too slight in degree to attract notice by the naked eye appearances of the specimen, may be detected, treated, and cured. Tuberculosis of the bladder, of that mild degree which is now being recognised more and more frequently, may be discovered in its early and curable stage in some cases in which nucleoproteid is in excess. By far the most common conditions, however, which give rise to this excess of nucleoproteid in the urine are those in which crystals are the cause of irritation. Phosphaturia is an example; the phosphates are more often amorphous than crystalline, it is true, but the stellar crystals of calcium phosphates may be abundant, and the very large prismatic crystals of magnesic phosphate (distinct from those of ammonio-magnesic phosphate) may be very irritating. Uric acid is another example; either the crystals themselves are the irritant, or else it is the general condition of those urines in which uric acid crystals are precipitated that is the cause of the trouble. Finally, oxaluria is perhaps the most potent cause of all in producing excess of nucleoproteid in urines. When calcium oxalate crystals are abundant, nucleoproteid is almost always abundant too; and the effects of the crystals in irritating the urinary passages are further indicated by the excess of leucocytes and the occasional red blood corpuscles that are usually detected by the microscope at the same time; in males, moreover, the urine may be full of spermatozoa in such cases.

Nucleoproteid, therefore, has a very different significance to albumen; it is certainly less important, and in many cases it has no importance at all; on the other hand, it may be of significance in leading to the early detection of serious lesions of the urinary passages, or it may be associated with crystals in the urine and irritation which may be more or less easily controlled by drugs or by modifications in the diet. In any case, just as with albuminuria, the presence of even a slight excess of nucleoproteid should lead to a careful examination of the urinary deposit under the microscope.

### THE EFFECTS OF MOTORING ON HEALTH.

Dr. Legendre, who has recently studied the effects of motoring on the health of the motorist, has communicated the results of his investigations to a French medical society. He finds that the immediate result of a motor drive is a considerable amount of vaso-constriction, followed by an equally appreciable vaso-dilatation and consequent fall in blood-pressure. Arguing from this, he observes that "the automobile may prove of service in the treatment of many disorders." In cases of emphysema, asthma, chlorosis, cardiac affections, and nervous depression, he has found motoring in moderation decidedly beneficial. He recommends it in cases of phthisis, provided the patient is not feverish. An interesting observation is that cardiac trouble, associated with atonic dyspepsia and chronic constipation, is much ameliorated by a course of motoring, while cases of gastric ulcer or organic disease of the stomach are never benefited.

